# Xylazine-induced diuresis in rats is attenuated by κ-opioid receptor and α2-adrenoceptor antagonists

**DOI:** 10.1007/s00210-026-05565-6

**Published:** 2026-06-17

**Authors:** Saadet Inan, Scott M. Rawls

**Affiliations:** 1 Center for Substance Abuse Research, Lewis Katz School of Medicine, Temple University, 3500 North Broad Street, Philadelphia, PA 19140 USA; 2 Department of Neural Sciences, Lewis Katz School of Medicine, Temple University, 3500 North Broad Street, Philadelphia, PA 19140 USA

**Keywords:** Xylazine, Diuresis, κ-Opioid receptor, α2-Adrenoceptors

## Abstract

Xylazine is an α2 adrenoceptor agonist approved for use in veterinary medicine as an analgesic and sedative. However, the recent use of xylazine as adulterant with opioids has created a public health emergency due to an increased number of addiction-related deaths and adverse effects. As a result, xylazine’s pharmacological profile has been revisited, and it has been found to act as a full agonist at κ-opioid receptors. Because increased urine output is a well-established effect of κ-opioid receptor agonists, we determined if κ-opioid receptors are involved in xylazine-induced diuresis. Adult male Sprague–Dawley rats were used. A dose–response curve was first established through systemic administration of xylazine. Rats were injected with xylazine (1.25, 2.5, or 5 mg/kg) or saline and then placed into metabolic cages for 2 h for urine collection. Xylazine doses of 2.5 and 5 mg/kg significantly increased urine output compared to saline-injected rats, indicating that xylazine caused diuresis. Next, rats were pretreated with saline; a κ-opioid receptor antagonist, 5′-guanidinonaltrindole (5′-GNTI) (0.01–0.1 mg/kg); or an α2-adrenoceptor antagonist, yohimbine (0.3–1 mg/kg). Thirty minutes later, rats were injected with saline or a fixed dose of xylazine (2.5 mg/kg) and urine was collected for 2 h. Pretreatment with either 5′-GNTI or yohimbine significantly decreased xylazine-induced diuresis. Our findings suggest that xylazine-induced diuresis is mediated by both κ-opioid receptors and α2-adrenoceptors, highlighting the importance of considering κ-opioid receptor activation when evaluating in vivo pharmacological effects of xylazine.

## Introduction

The pharmacological profile of xylazine is currently being revisited due to its use as an adulterant with illicit opioids and psychostimulants (Nunez et al. 2021; Mumba et al. 2023; Amaral et al. 2025; Dahlen et al. 2026). Xylazine is a structural analog of clonidine, another α2-adrenoceptor agonist that is approved by the FDA as an antihypertensive medication (Antonaccio et al. 1973). The use of xylazine as an adulterant goes back to the 2000 s in Puerto Rico (Torruella 2011) before spreading to North America where it was first detected in Philadelphia (Wong et al. 2008). Overdose deaths in the United States related to the combination of xylazine with opioids increased from 2019 to 2022 (Vohra et al. 2023; Zhu and Cano 2025), and The Office of National Drug Control Policy declared the xylazine/fentanyl combination as an Emergent Threat in 2023. Clinical and preclinical evidence about the impacts of xylazine on the pharmacological effects of opioids remains sparse. The addition of xylazine to fentanyl enhances and prolongs euphoria in human users (D'Orazio et al. 2023). Xylazine also causes hypothermia and brain hypoxia that exacerbates respiratory depression and mortality caused by fentanyl (Demery et al. 2025; Choi et al. 2023). In preclinical studies, xylazine also worsens opioid-induced respiratory depression in rodents (Demery et al. 2025; Choi et al. 2023). Yet, contrary to human reports, rodent studies show that xylazine does not enhance rewarding and motivational effects of opioids (Acosta-Mares et al. 2023; St Onge et al. 2024; Hochstetler et al. 2025; Samini et al. 2008; Khatri et al. 2024).

Understanding xylazine–opioid interactions requires a clearer definition of xylazine’s pharmacological profile. Beyond activation of α2-adrenoceptors, xylazine also affects other receptor targets, as demonstrated by Bedard et al. (2024). In radioligand binding assays, xylazine inhibited binding by ≥ 50% at α2-adrenoceptors, κ-opioid receptors, 5-HT7 serotonin receptors, and σ1 and σ2 receptors (Bedard et al. 2024). In a PRESTO-tango GPCRome screen for potential agonist activity at human GPCRs, xylazine activated α2-adrenoceptors, κ-opioid receptors, and dopamine D2 receptors (Bedard et al. 2024). Xylazine also displaced a radiolabeled κ-opioid receptor agonist 3H-U69593 and showed activity on G_i1_ and G_oA_ and G_z_ pathways but no activity on ß-arrestin 1 or 2 recruitment, suggesting biased agonism at κ-opioid receptors (Bedard et al. 2024). A computational study using docking and molecular dynamics simulations showed that xylazine engages binding-site residues within the κ-opioid receptor, identifying specific interaction motifs that may underline receptor affinity (Floresta et al. 2025). Given the identification of xylazine as a full agonist at κ-opioid receptors, we used a standard diuresis assay in rats to investigate in vivo κ-opioid receptor activity of xylazine. Diuresis is defined as an increase in urine output, and systemic administration of κ-opioid receptor agonists induce robust diuresis (Craft et al. 2000; Inan et al. 2009; Meariman et al. 2022). Our results show that xylazine induces dose-dependent diuresis that is attenuated by κ-opioid receptor or α2-adrenoceptor antagonism.

## Materials and methods

### Chemicals

Xylazine was obtained from Covetrus Inc (Portland, ME, USA) as 100 mg/ml solution and diluted with sterile saline. 5′-guanidinonaltrindole (5′-GNTI) and yohimbine hydrochloride were purchased from Sigma-Aldrich (St. Louis, MO, USA) and dissolved in sterile saline.

### Experimental design

#### Animals

The study was conducted with 122 adult male Sprague–Dawley rats (250–275 g; *n* = 5–10; Envigo Laboratories, Denver, PA, USA). The rats were housed two per cage with free access to food and water in the Animal Facility for at least 4 d. A standard light/dark cycle was maintained with a timer-regulated light period from 7:00 AM to 7:00 PM. Experiments were conducted between 10:00 AM and 5:00 PM. Experimental procedures were approved by the Temple University Institutional Animal Care and Use Committee. On the experimental day, rats were brought to the laboratory and acclimated for at least 1 h with free access to food and water before injections.

### Diuresis study

To determine if systemically administered xylazine induces diuresis, rats were injected intraperitoneally (IP) with saline or xylazine (1.25, 2.5, or 5 mg/kg) and placed into individual metabolic cages with no access to food and water for urine collection over 2 h as described previously (Inan et al. 2009). Urine collection time was chosen based on pharmacokinetic of xylazine (Veilleux-Lemieux et al. 2013). Urine was collected in graduated cylinders, and total urine volume was recorded. Next, based on the xylazine dose-response curve, a submaximal dose of xylazine was chosen to study a role for κ-opioid receptors and α2-adrenoceptors in xylazine-induced diuresis. Rats were injected subcutaneously (SC) with 5′-GNTI (0.01, 0.03, or 0.1 mg/kg), yohimbine (0.3 or 1 mg/kg), or saline. Thirty min later, rats were injected with a fixed dose of xylazine (2.5 mg/kg) or saline and placed into cages for 2 h for urine collection.

### Statistical analysis

Data were analyzed by one-way analysis of variance (ANOVA) followed by Dunnett’s multiple comparison or two-way ANOVA (pretreatment × xylazine treatment) followed by Newman-Keuls multiple comparison tests. GraphPad Prism version 10.1.2 was used for statistical analysis. Data were expressed as mean ± standard error of mean (S.E.M.), and *p* < 0.05 was considered statistically significant.

## Results

**Fig. 1:**
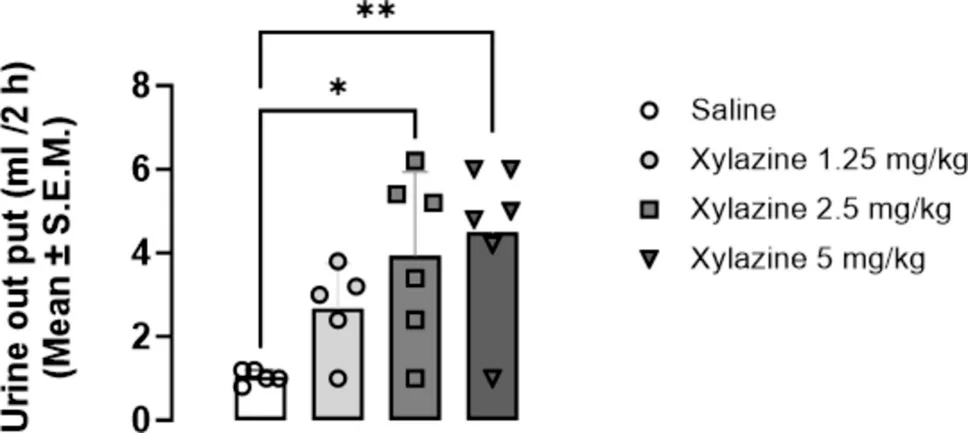
Xylazine increased urine output in a dose-dependent manner. Rats were injected with saline or xylazine (1.25, 2.5, or 5 mg/kg, IP) and placed into metabolic cages for 2 h for urine collection. *p < 0.05 or **p < 0.01 compared to saline *n* = 5–6

**Fig. 2:**
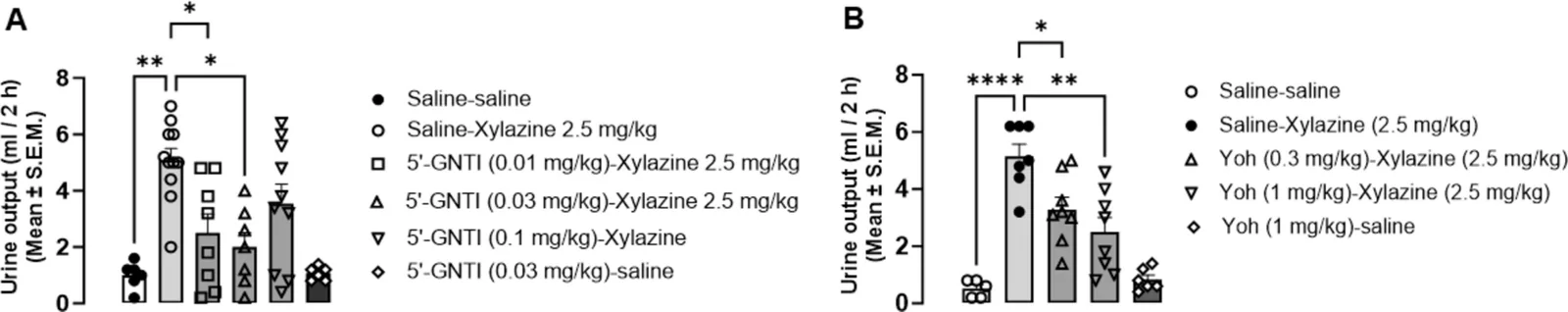
Pretreatment with a κ-opioid receptor antagonist (5՛-GNTI) or α2-adrenoceptor antagonist (yohimbine) reduced diuresis induced by xylazine. (A) 5′-GNTI (0.01 or 0.03 mg/kg) reduced xylazine-induced diuresis. **p < 0.01 compared to drug-naïve rats (Saline-saline) and *p < 0.05 compared to Saline-xylazine (2.5 mg/kg), *n* = 6–10. (B) Yohimbine (0.3 or 1 mg/kg) reduced xylazine-induced diuresis. ****p < 0.0001 compared to drug-naïve rats (Saline-saline) and *p < 0.05 or **p < 0.01 compared to Saline-xylazine (2.5 mg/kg), *n* = 6–10

Xylazine induced a dose-dependent diuresis (Fig. 1). One-way ANOVA revealed a significant main effect ([F (3, 18) = 5.432], *p* = 0.0077). Xylazine at doses of 2.5 and 5 mg/kg significantly increased urine output compared to saline (*p < 0.05 and **p < 0.01, respectively). In rats treated with 1.25 mg/kg of xylazine, urine output was not significantly different than saline-treated rats (*p* > 0.05). Pretreatment with 5′-GNTI at doses of 0.01 and 0.03, but not 0.1 mg/kg, significantly reduced xylazine-induced diuresis (Fig. 2A). Two-way ANOVA showed a significant effect of xylazine treatment ([F (4, 26) = 5.082], *p* = 0.0037). As expected, rats treated with xylazine by itself significantly increased urine output compared to drug-naïve rats (**p < 0.01). However, in rats treated with xylazine, pretreatment with 5′-GNTI at doses of 0.01 or 0.03 mg/kg significantly reduced urine output compared to saline pretreatment (*p < 0.05). In rats that received 5′-GNTI (0.03 mg/kg) by itself, urine output was not significantly different compared to drug-naïve rats (Fig. 2A).

Yohimbine pretreatment also significantly reduced the increased urine output caused by xylazine, with two-way ANOVA showing a significant effect of xylazine treatment ([F (3, 17) = 12.90], *p* = 0.0001) (Fig. 2B). Xylazine treatment by itself again increased urine output compared to rats that were entirely drug-naïve (****p < 0.0001). In rats treated with xylazine, urine output was significantly reduced by pretreatment with yohimbine (0.3 or 1 mg/kg) compared to pretreatment with saline (*p < 0.05, **p < 0.01, respectively). Additionally, no difference in urine output was detected between rats that received yohimbine (1 mg/kg) by itself compared to drug-naïve rats (Fig. 2B).

## Discussion

We provide pharmacological evidence that xylazine causes diuresis in rats that is dependent on active κ-opioid receptors and α2-adrenoceptors. Xylazine elicited diuresis in a dose-related manner, and the diuresis was inhibited by pretreatment with either a κ-opioid receptor antagonist or α2-adrenoceptor antagonist. Previously, it was reported that clonidine and other α2-adrenoceptor agonists induce diuretic effects in rats and the effect is through activation of α2-adrenoceptors but not α1-adrenoceptors (Miller 1980; Shockley et al. 1993). Diuretic effects of xylazine in rats, cats and dogs were also shown previously, and the effect was diminished by an α2-adrenoceptor antagonist but not by an α1-adrenoceptor antagonist (Mohammad et al. 1989; Miller et al. 2001; Talukder et al. 2009; Murahata et al. 2014).

Our study is the first to demonstrate that κ-opioid receptor activity contributes to the diuretic effects of xylazine. We used a highly selective and potent κ-opioid receptor antagonist, 5′-GNTI, which in our previous report inhibited diuresis evoked by a battery of κ-opioid receptor agonists (nalfurafine, U50,488, and MOM-salvinorin B) (Inan et al. 2009). Having an inhibition with the lower doses (0.01 and 0.03 mg/kg) of 5′-GNTI, but no effect with a higher dose (0.1 mg/kg) of 5′-GNTI was surprising since 5′-GNTI at 0.1–3 mg/kg doses significantly reduced nalfurafine-induced diuresis in rats in our previous study (Inan et al. 2009). The lack of effect of a higher dose of 5′-GNTI on xylazine-induced diuresis can be potentially explained by functional interactions between κ-opioid receptor and α2-adrenoceptors. Indeed, there is evidence of functional interactions between these two systems. For example, in one of the earliest studies, it was shown that both the κ-opioid receptor agonist, ethylketocyclazocine (EKC), and clonidine individually depressed stimulation-induced noradrenalin release whereas yohimbine increased presynaptic noradrenalin release in rabbit cortical slices (Limberger et al. 1986). When slices were pretreated with clonidine and then treated with EKC, the EKC-induced effect on noradrenalin was reduced. On the other hand, when slices were pretreated with yohimbine, the EKC-induced effect was enhanced. Lastly, in slices pretreated with EKC and then treated with clonidine, the clonidine-induced reduction of noradrenalin release was attenuated. It was concluded that presynaptic α2-adrenoceptors and κ-opioid receptors interact, either directly at the receptor level or through their downstream signaling pathways. Activation of one receptor system lessened the inhibition of release produced by activation of the other receptor system. Similar results were also reported with rabbit hippocampal slices (Allgaier et al. 1989). In that study, yohimbine also reduced EKC-induced presynaptic noradrenaline release, suggesting that the two receptor systems compete for a common pool of G proteins.

It was beyond the scope of our study to determine whether the diuretic effect of xylazine is mediated centrally, peripherally, or both; whether it occurs through inhibition of arginine vasopressin (AVP), also known as antidiuretic hormone (ADH); or whether the diuresis reflects increased water excretion alone or also involves sodium and potassium loss. κ-opioid receptor agonist-induced diuresis is characterized by an increase in urine flow as well as a decrease in urine osmolality without an associated increase in Na + excretion, suggesting a water diuresis (VonVoigtlander et al. 1983; Ashton et al. 1989; Yamada et al. 1989; Gadano et al. 2000; Gottlieb et al. 2005; Inan et al. 2009). Both centrally and peripherally acting κ-opioid receptor agonists promote diuresis (Barber et al. 1994; Inan et al. 2009; Beck et al. 2019; Meariman et al. 2022). Systemic and intracranial administrations of κ-opioid receptor antagonists attenuate κ-opioid receptor agonist induced-diuresis, suggesting both peripheral and central activation (Inan et al. 2009; Meariman et al. 2022). Preclinical studies also reported that ADH secretion and release are diminished by κ-opioid receptor agonists (Slizgi and Ludens 1982, 1986; Leander et al. 1985; Yamada et al. 1989). On the other hand, it was reported that ADH release was not affected in clonidine-induced diuresis (Miller 1980; Leander et al. 1985). However, one of the earliest studies reported that iv administration of clonidine in dogs induced water diuresis with increased sodium excretion (different from κ-opioid receptor activation induced water diuresis), increase in plasma renin levels as well as increase in prostaglandin E excretion in urine and reduction in diuresis in response to ADH infusion, suggesting more peripheral mechanism and ADH involvement in the α2-adrenoceptor activation-induced diuresis (Olsen 1976). Olsen (1976) also reported no increase of secretion of potassium and chloride in the urine.

Further mechanistic studies can be performed to explore the contribution of κ-opioid receptor activation to xylazine-induced diuresis. For example, determining effects of intracranial injections of 5′-GNTI or yohimbine on xylazine-induced diuresis would provide more knowledge on whether central inhibition of κ-opioid receptor or α2-adrenoceptors reduces xylazine-induced diuresis. Determining changes in plasma and urine AVP levels, as well as urine osmolality and electrolyte levels, could also provide useful mechanistic insights. However, since decreases in AVP levels and water diuresis are also features of α2-adrenoceptor activation-induced diuresis, determining these effects would not discriminate a κ-opioid receptor or α2-adrenoceptor mechanism for xylazine.

In conclusion, we showed for the first time that xylazine-induced diuresis involves κ-opioid receptor and α2-adrenoceptor activation. Because our results demonstrate that κ-opioid receptor activation contributes to xylazine’s pharmacological profile, both κ-opioid receptors and α2-adrenoceptors should be considered in elucidating its effects in the context of illicit substance misuse (Bedard et al. 2024).

## Data Availability

All source data for this work (or generated in this study) are available upon reasonable request.
